# Which technique of positioning and immobilization is better for breast cancer patients in postmastectomy IMRT, single‐pole or double‐pole immobilization?

**DOI:** 10.1002/acm2.12506

**Published:** 2018-12-03

**Authors:** Qun Xiang, Wuyun Jie, KuiKui Zhu, Qiong Wang, Jing Cheng

**Affiliations:** ^1^ Cancer Center Union Hospital Tongji Medical College Huazhong University of Science and Technology Wuhan China; ^2^ Affiliated Tumor Hospital of Xinjiang Medical University Urumuqi China

**Keywords:** conformity index, homogeneity index, postmastectomy IMRT, radiotherapy immobilization

## Abstract

**Purpose:**

Our purpose was to explore which immobilization is more suitable for clinical practice in postmastectomy intensity modulation radiotherapy, the single‐pole position or the double‐pole position?

**Methods:**

Patients treated with postmastectomy intensity modulation radiotherapy were eligible. They were selected randomly for single‐pole position or double‐pole position. Dose–volume histogram (DVH) was used to evaluate plans. After their first radiotherapy, the physicians asked a question about the comfort level of their position. The dosimetric parameters, comfort levels, and reproducibility of the two immobilization techniques were collected and analyzed after all patients had finished the whole radiotherapy.

**Results:**

Totally, 94 patients were enrolled. Of these, 54 patients were treated with the single‐pole position, 28 (51.9%)had left‐sided lesions. While 40 patients were treated with the double‐pole position, 20 (50%) had left‐sided lesions. Patients’ characteristics in two groups were comparable. The single‐pole and double‐pole immobilizations had similar conformity (0.60 ± 0.05 vs 0.60 ± 0.06, *P* = 0.887) and homogeneity index (0.14 ± 0.03 vs 0.13 ± 0.03, *P* = 0.407). Compared to single‐pole position, double‐pole position typically increased the mean dose, *V*_20_, and *V*_30_ of heart (*P* < 0.05). Moreover, patients in the single‐pole group felt more comfortable than another group (*P* < 0.05). There was no difference in reproducibility between the two groups (*P* > 0.05).

**Conclusions:**

Single‐pole position seems to be more comfortable and can reduce dose coverage to heart. Both devices allow for reproducible setup and acceptable dosimetry.

## 1 INTRODUCTION

**Figure 1:**
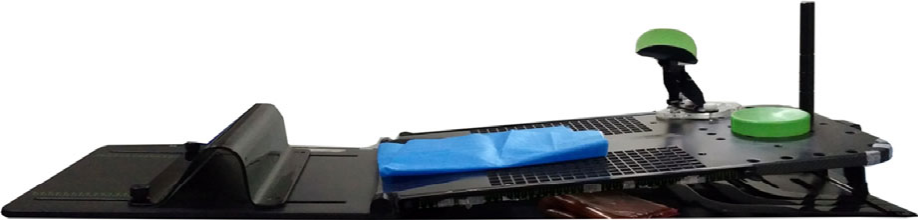
The breast bracket.

Breast cancer is the most common cancer in Chinese women.^1^ Postmastectomy intensity modulation radiotherapy (IMRT) for breast cancer patients is a very mature radiotherapy technique, and it can reduce the local recurrence rate and improve the overall survival rate.^2^ With the advent of the era of precise radiotherapy, IMRT can potentially increase the coverage of the target volume and reduce the nonconformity of the dose distribution.^2^ More importantly, IMRT can reduce the dose delivered to organs at risk and can reduce complications in patients receiving extensive regional radiotherapy.^3^, ^4^ It is critical to fix the position of the patient throughout radiotherapy. To reproduce patient position and minimize their movement during treatment, the immobilization device must be supportive and comfortable. The breast bracket (Fig. 1) has become the main beneficial tool for immobilization of the breast during treatment. Either the single‐pole or double‐pole position can be used in our clinical work. However, regarding the issue of which position is more appropriate, there is no relevant research comparing them. Our study collected the dosimetric parameters of 94 cases of breast cancer patients who had been treated with IMRT in our department after radical mastectomy from January 2015 to September 2016. By comparing the radiotherapy dosimetric parameters, comfort levels and the reproducibility of two groups, our study analyzed which position is more suitable for clinical practice.

## 2 MATERIALS AND METHODS

### 2.A Patient information

Breast cancer patients who received postmastectomy IMRT were involved in our study from January 2015 to September 2016. The exclusion criteria were as follows: (a) distant metastases at diagnosis; (b) male breast cancer patients; (c) history of other malignancies. A total of 94 patients were eligible for our study. Patients’ age ranged from 30 to 66 yr, and stages II and III disease were presented. Of these, 54 patients were treated with the single‐pole position, 28 (51.9%) had left‐sided lesions. While 40 patients were treated with the double‐pole position, 20 (50%) had left‐sided lesions. Patients’ characteristics of two groups are shown on Table 1. All patients had IMRT plans approved by treating physicians. Written informed consents were collected prior to treatment procedure. The study was performed according to a protocol approved by the Huazhong University of Science and Technology Institutional Ethics Committee.

**Table 1:** Patients’ characters

Parameters	Single‐pole	Double‐pole	*P* value
No. of patients	54	40	–
Median age, years (range)	50.5(30–66)	50(30–66)	0.836
Mean weight (Kg)	58.81 ± 8.11	56.5 ± 8.05	0.177
Treated side			
Left	28	20	0.859
Right	26	20	

### 2.B Radiotherapy

All patients underwent standard exposure and were supinated on the breast bracket with both arms extended above their head. Their heads were turned to the contralateral side of the affected breast as much as they could.

**Figure 2:**
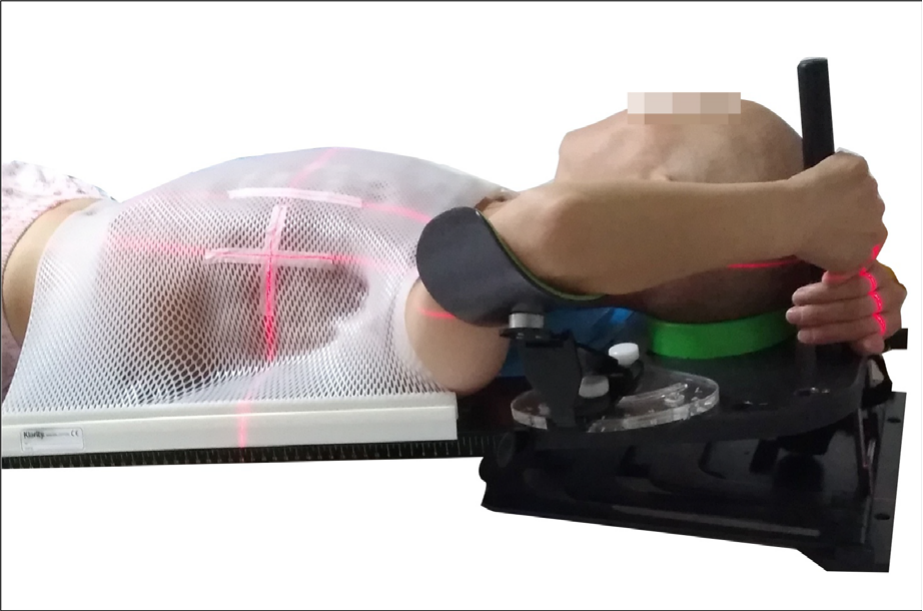
Single‐pole position (1) The single pole was on the ipsilateral side of the head; (2) The arm on the treatment side abducted to 90° with both hands gripping the single pole; (3) The ipsilateral upper limb gripping the pole over the contralateral upper limb.

#### 2.B.1 Single‐pole position (Fig. 2)

1. The single pole was on the ipsilateral side of the head.
2. The arm on the treatment side abducted to 90° with both hands gripping the single pole.
3. The ipsilateral upper limb gripping the pole over the contralateral upper limb.

#### 2.B.2 Double‐pole position

**Figure 3:**
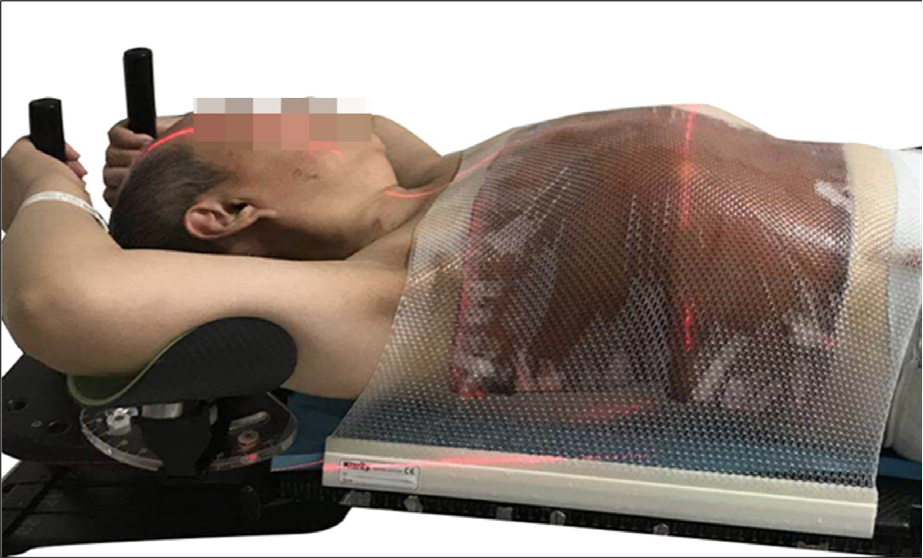
Double‐pole position each side of the head has a pole, with each hand gripping one pole.

Each side of the head had a pole, with each hand gripping one pole (Fig. 3).

Hoses filled with computed tomography (CT) contrast agent were used to mark the caudal border, lateral border, and the cranial border of the target volume, as well as the mastectomy scar area. The caudal border was defined as 1 cm below the margin of the contralateral mammary gland. The lateral border was at the mid‐axillary line. The cranial border of the chest wall skin was the infraclavicular edge. The ipsilateral chest wall below the clavicle was covered with a 5 mm thermoplastic mold to reduce setup errors.

A large‐aperture CT‐simulation was performed in the treatment position on the breast bracket. The CT scan was performed using a 5‐mm slice thickness, with scanning range running from the base of the skull to the lower edge of the liver. The CT images were exported to the Pinnacle 9.2 Treatment Planning System for clinical contouring of target volume. The clinical target volume (CTV) was defined to consist of ipsilateral chest wall, mastectomy scar, the supraclavicular and infraclavicular lymphatic drainage areas. Each CTV of the chest wall and regional lymph node were delineated according to breast cancer atlas for radiation therapy planning consensus definitions of the Radiation Therapy Oncology Group (RTOG).^5^ The borders of the CTV for chest wall were as follows: (a) the cranial border was marked at inferior border of the clavicular head; (b) the caudal border was marked at the contralateral inframammary fold; (c) the anterior border was 2 mm below the skin surface; (d) the posterior border was rib‐pleural interface; (e) the lateral border was at the mid‐axillary line; (6) the medial border was the ipsilateral sternocostal junction. The chest wall CTV was expanded by 5 mm to construct planning target volume (PTV), while still conserving 2 mm under the skin surface integrity. Organs at risk (OARs) including bilateral lungs, heart and spinal cord, and contralateral breast were contoured. The heart was contoured along with pericardial sac. The superior aspect (or base) of the contour began at the level of the inferior aspect of the pulmonary artery passing the midline and extended inferiorly to the apex of the heart.^6^

Our department utilized a multiple beam integrated plan, and a simplified IMRT plan was generated using Pinnacle treatment planning software (version 9.2). All plans were optimized to cover the entirety of the PTVs and spare surrounding normal tissues as much as possible. For the purpose of improving skin sparing dose and avoiding calculation errors of a dose built‐up area, a daily 5‐mm bolus was placed on the chest wall of each patient. The optimization process started with dose–volume constraints such as: (a) 95% of PTV receiving 50 Gy in 25 fractions; (b) the percent volume of PTV receiving 110% prescription dose was ≤5%; (c) ≤1% of the spinal cord received ≤40 Gy; (d) ≤30–35% of the ipsilateral lung was exposed to ≤20 Gy; (e) ≤20% of the total lung got ≤20 Gy; (f) the heart mean dose remaining at ≤10 Gy for left‐sided lesions and ≤6 Gy for right‐sided lesions. The priority was high for PTV, heart, and lung constraints relative to other structures. We always restricted the dose to hearts and lungs under the dose–volume constraints first, and then guaranteed the PTV dose next. Optimization proceeded until no further improvement was seen. All treatments were delivered with 6‐MV photon beams using a VARIAN UNIQUE‐SN2236 linear accelerator. Verification was applied on a weekly basis.

### 2.C Conformity index (CI)

For determination of the conformity index of PTV, we used the following definition:

$$\text{CI}=\left(V_{\mathrm{Tref}}/\text{VT}\right)\times \left(V_{\mathrm{Tref}}/V_{\mathrm{ref}}\right)$$

where *V*_Tref_ is the target volume covered by the 95% isodose line, *V*_ref_ is the treated volume covered by the 95% isodose line and VT is the volume of target. The CI value range is 0–1, the greater value means the better conformity.^7^

### 2.D Homogeneity index (HI)

To determine the homogeneity index of PTV, we used the following equation

$$\text{HI}=\frac{D_{2}-D_{98}}{D_{\mathrm{median}}}$$

where *D*_median_ is the median dose to the TV, D_2_ and D_98_ are the maximum and minimum dose that covers 2% and 98% volume of the PTV on dose–volume histogram. Lower HI correlates with a more homogeneous target dose.^8^

### 2.E Comfort levels

Comfort refers to a sense of being in a physically and spiritually healthy and peaceful state. It means individual's body and mind are relaxed and satisfied, with no anxiety nor pain.^9^ Comfort includes physical comfort, psychological comfort, social and cultural comfort, and a comfortable environment.^10^ In some previous studies on comfort levels, researchers used visual analog scores to assess the patients’ comfort levels. Hamilton evaluated patients’ comfort by using a visual analog assessment method in a comfort study of 14 patients with advanced cancer.^11^ However, no studies have been done regarding the comfort levels of breast cancer patients during radiotherapy immobilization. In this study, the physician asked patients a question about the comfort levels of their radiotherapy position after first treatment. (Question: Did you feel comfortable during radiotherapy at this immobilization unit? A. comfortable, B. uncomfortable.) We collected the answers of the two groups and compared the differences in their respective comfort zones.

### 2.F Reproducibility

When patients were first treated, they were immobilized according to the positioning mark provided by the CT simulator. An electronic portal imaging device (EPID) was used each week for real‐time verification, and data about the patients’ left‐right (*X*), craniocaudal (*Y*), and ventrodorsal (*Z*) setup deviations were collected for analysis. We adopted the coordinate system used in the report ICRU 62, in which the *X*‐axis represents the left and right direction, the *Y*‐axis represents the cranial and caudal direction, and the *Z*‐axis indicates the anterior and posterior direction.^12^ The anterior, right, and caudal directions were defined as positive values, while the posterior, left, and cranial directions were defined as negative values.^12^ Total 3D vector error was defined using the formula:^13^

$$\text{Total}=\sqrt{X^{2}+Y^{2}+Z^{2}}$$

The larger the value of the total 3D vector, the greater would be the overall setup displacement.^14^

### 2.G Plan evaluation

For dosimetric analysis, the following parameters extracted from dose–volume histograms (DVHs) were used:

1. *V*_55_, *D*_min_, *D*_max_, and *D*_mean_ for PTV: *V*_55_ was defined to be the percent volume receiving 55 Gy of PTV; *D*_min_ was the minimal dose to the PTV; while *D*_max_ was the maximal dose to PTV. The *D*_mean_ was the mean dose at PTV.
2. The ipsilateral lung *V*_5_, *V*_20_, and *D*_mean_ were: *V*_5_, *V*_20_ defined as the percent volume receiving 5 Gy and 20 Gy of the ipsilateral lung, respectively; *D*_mean_ was the mean dose to ipsilateral lung.
3. The contralateral lung *V*_5_ and *D*_mean_: where *V*_5_ was defined to be the percent volume receiving 5 Gy at the contralateral lung, and *D*_mean_ was the mean dose delivered to the contralateral lung.
4. The heart *V*_5_, *V*_10_, *V*_20_, *V*_30_, *D*_mean_: the heart *V*_5_, *V*_10_, *V*_20_, *V*_30_ were defined to be the percent volume receiving 5 Gy, 10 Gy, 20 Gy, 30 Gy of the heart, respectively; and *D*_mean_ was the mean dose to the heart.
5. Whole lung *V*_5_, *V*_20_, and *D*_mean_: *V*_5_, *V*_20_ were defined to be the percent volume receiving 5 Gy and 20 Gy of the whole lung, respectively. The *D*_mean_ was calculated by mean dose to the whole lung.
6. The spinal cord *D*_max_ was determined by maximal dose to the whole length spinal cord.

### 2.H Statistical methods

In this study, statistical analyses were performed by SPSS 18.0 (SPSS, Chicago, IL) software. (a) Two independent samples *t*‐test was applied to measure the volume‐dose of the two groups, including PTV (*D*_min_, *D*_max_, *D*_mean_), heart (*V*_5_, *V*_10_, *D*_mean_), ipsilateral lung (*V*_5_, *D*_mean_), whole lung (*V*_5_, *V*_20_, *D*_mean_), and the spinal cord (*D*_max_). (b) Nonparametric rank sum test was applied to analyze the volume–dose relationship of CI, HI, reproducibility, PTV (*V*_55_), heart volume (*V*_20_, *V*_30_), ipsilateral lung volume (*V*_20_), and contralateral lung (*V*_5_, *D*_mean_) between the two groups. (c) The chi‐square test was used to compare the comfort levels of the two groups. All measurement data were expressed as mean ± standard deviation (mean ± SD), *P* < 0.05 were considered statistically significant.

## 3 RESULTS

Patients’ characteristics of two groups and the proportion of left‐sided and right‐sided lesions per group (Table 1) have no significant differences, *P* > 0.05.

### 3.A Target coverage and homogeneity

Table 2 summarized the results of PTV dosimetry. In comparison with single‐pole position, double‐pole position had higher *D*_min_. However, their dose conformity and homogeneity were similar. In addition, the *V*_55_ was generally <5%, indicating that a very small volume of PTV received 110% of prescription dose.

**Table 2:** Summary of DVH‐based analysis for the PTV

Parameters	Single‐pole	Double‐pole	*P* value
*V*_55_ (%)	2.33 ± 2.39	2.80 ± 2.51	0.276
*D*_min_ (cGy)	3552.31 ± 544.12	3793.41 ± 385.22	0.019*
*D*_max_ (cGy)	5730.54 ± 120.93	5744.31 ± 115.11	0.579
*D*_mean_ (cGy)	5173.82 ± 80.25	5193.01 ± 44.61	0.176
CI	0.60 ± 0.05	0.60 ± 0.06	0.887
HI	0.14 ± 0.03	0.13 ± 0.03	0.407

DVH, dose–volume histogram; PTV, planning target volume; *V*_55_, the percentage of PTV volume that received 55 Gy in the total PTV; *D*_min_, the minimum dose of PTV; *D*_max_, the maximum dose of PTV; *D*_mean_, average dose of PTV; CI, conformity index; HI, homogeneity index.

*The difference was statistically significant (*P* < 0.05).

### 3.B OARs

Table 3 listed the dose–volume statistics of OARs. All IMRT plans were clinically acceptable regarding the dose–volume of heart, lung, and spinal cord irradiated. As compared to single‐pole position, double‐pole position typically increased the mean dose (*D*_mean_) (*P* = 0.006), *V*_20_ (*P* = 0.001), and *V*_30_ (*P* = 0.001) of heart. Other dosimetric parameters had no significant differences between two groups.

**Table 3:** Summary of DVH‐based analysis for OARs

	Parameters	Single‐pole	Double‐pole	*P* value
Heart	*V*_5_ (%)	42.96 ± 22.13	45.12 ± 17.11	0.594
	*V*_10_ (%)	17.85 ± 11.99	22.40 ± 10.53	0.059
	*V*_20_ (%)	6.70 ± 6.15	11.02 ± 6.08	0.001*
	*V*_30_ (%)	3.17 ± 3.28	6.15 ± 4.13	0.001*
	*D*_mean_ (cGy)	657.52 ± 299.67	834.12 ± 305.48	0.006*
Ipsilateral lung	*V*_5_ (%)	59.27 ± 5.08	59.23 ± 5.60	0.967
	*V*_20_ (%)	31.77 ± 2.63	30.89 ± 5.71	0.267
	*D*_mean_ (cGy)	1644.45 ± 129.42	1638.72 ± 116.56	0.823
Contralateral lung	*V*_5_ (%)	4.46 ± 8.04	4.46 ± 8.04	0.997
	*D*_mean_ (cGy)	131.64 ± 71.97	128.17 ± 49.37	0.921
Whole lung	*V*_5_ (%)	33.92 ± 7.96	32.11 ± 5.26	0.215
	*V*_20_ (%)	17.01 ± 3.80	15.84 ± 2.43	0.072
	*D*_mean_ (cGy)	965.36 ± 258.16	905.29 ± 209.36	0.231
Spinal cord	*D*_max_ (cGy)	1996.15 ± 693.52	2221.62 ± 722.64	0.129

OARs, organs at risk; *V*_x_, percent volume of critical structures receiving a dose of x Gy; *D*_mean_, mean dose.

*The difference was statistically significant (*P* < 0.05).

### 3.C Reproducibility

Table 4 presented setup deviations of left‐right (*X*), craniocaudal (*Y*), ventrodorsal (*Z*), and total 3D vector error. There was no difference in *X*,* Y*,* Z*, and total 3D vector error between the two groups, *P* > 0.05.

**Table 4:** Reproducibility

Parameters	Single‐pole	Double‐pole	*P* value
*X* (cm)	0.07 ± 0.17	0.05 ± 0.18	0.463
*Y* (cm)	0.04 ± 0.26	0.02 ± 0.18	0.540
*Z* (cm)	−0.07 ± 0.19	−0.07 ± 0.14	0.996
Total	0.30 ± 0.23	0.26 ± 0.15	0.313

*X*, left‐right; *Y*, craniocaudal; *Z*, ventrodorsal; Total, total 3D vector error.

### 3.D Comfort levels

As for comfort levels, the single‐pole position group had a higher comfort rate (81.5%) than that in double‐pole position group (37.5%) (*P* < 0.05, Table 5).

**Table 5:** Comfort state

Group	Comfort state		Comfort rate	*X* ^2^	*P*
	Comfortable	Uncomfortable			
Single pole	44	10	81.5% (44/54)	19.020^a^	0.000
Double pole	15	25	37.5% (15/40)		

a 0 cells (.0%) have expected count less than 5. The minimum expected count is 14.89.

## 4 DISCUSSION

With the development of precision radiotherapy, the goal of radiotherapy is minimizing the risk of normal tissue damage while delivering a dose distribution that will result in a high level of local control.^15^ Deep inspiration breath hold (DIBH) is an effective technique to reduce cardiac and pulmonary dose during breast radiotherapy, but it is expensive and has technical challenges of program implementation; therefore, it has not been widely adopted in clinical practice.^16^ Intensity‐modulated radiation therapy (IMRT), as one of techniques for cardiac protection/avoidance, can decrease the mean dose, maximum dose, *V*_5_, *V*_20_, *V*_30_ of heart.^17^ Furthermore, IMRT causes reduction in cardiac normal tissue complication probabilities (NTCP) compared with three‐dimensional conformal radiotherapy (3D‐CRT).^17^ Our department adopted postmastectomy IMRT for breast cancer radiotherapy. There are two different positions by using the breast bracket device. In our study, patients’ characteristics of two groups were comparable. We found that both single‐pole and double‐pole positions allowed for reproducible setup. At the same time, the *V*_20_, *V*_30_, and *D*_mean_ of heart in the single‐pole group were smaller than those of double‐pole position. Even so, both positions could offer acceptable dosimetry for patients. With the increase in dose delivering to the heart, the risk of subsequent heart disease raised.^18^ Therefore, the single‐pole position might decrease the risk of subsequent heart disease compared with the double‐pole position.

There are publications trying to have predictors of heart dose with various measurements.^19^, ^20^ Investigators used the cranial–caudal distance of the heart in contact with the anterior chest wall or the “4th Arch” metric to explore the influence of anatomic features in women at risk of cardiac exposure from whole breast radiotherapy. Therefore, the anatomy factors might affect the heart dose in the two positions. We would like to bring the anatomy features in our further study. However, our conclusion might be just a random result in the double‐pole position, and further studies are needed to confirm our result.

In addition to the radiation dose and the reproducibility, the comfort levels of patients are also important. The diagnosis of breast cancer and the followed breast cancer treatments always made the patients felt anxious. Radiotherapy in itself is a stress factor for patients.^21^ The first factor affecting patient compliance is anxiety, and the second one is physical discomfort. The more comfortable the patients felt, the lower levels of anxiety the patients presented. So we also paid attention to identify the relationship between the setup devices and the patients’ comfort. In Kolcaba's study, they used guided imagery to increase patients’ comfort. Then, they designed a tool to measure the comfort levels of early breast cancer patients who underwent radiotherapy.^22^ The table was named the radiotherapy comfort scale, but it has not been used widely. In our study, patients were asked their feelings under treatment position after their first treatments. This could help us to exclude other factors such as the side‐effects of radiotherapy, ongoing treatment and the adaptability of patients, which could in turn affect the comfort levels of the patients. Because their degree of anxiety might vary greatly with the treatments numbers increasing, this affected the comfort levels during treatment procedure. Moreover, economic conditions and cultural education varied between patients, this could influence the comfort extent. The results of our study suggested that the single‐pole position is more comfortable than the double‐pole position. Apart from the comfort level of the posture itself, there may be other factors which affected the patients’ subjective feelings during radiotherapy.

However, there are several limitations in this study. First, we did not record the time each position spent, thus we could not evaluate which position could reduce the setup time properly. Second, the sample size is small and large clinical trials are needed to verify our findings. Third, the study lacks a relevant authoritative scale that can more accurately evaluate the comfort zone. The evaluation used in this study could not exclude subjectivity totally. Fourth, this is a single‐institution study, and we did not collect patients’ methods to deal with discomfort. There may be cultural differences in coping with pain between different countries. Last but not least, variations in target volumes contouring and plans validation by physicians might affect the dosimetry of PTV and OAR. Therefore, further studies are needed to overcome these limitations.

## 5 CONCLUSION

For breast cancer patients in postmastectomy IMRT, the single‐pole position seems to be more comfortable and can reduce dose coverage to heart. Both devices could allow for reproducible setup and acceptable dosimetry. Further studies are needed to confirm our result.

## CONFLICT OF INTEREST

No conflicts of interest.
